# Gastrointestinal Dopamine in Inflammatory Bowel Diseases: A Systematic Review

**DOI:** 10.3390/ijms222312932

**Published:** 2021-11-29

**Authors:** Magdalena Kurnik-Łucka, Paweł Pasieka, Patrycja Łączak, Marcin Wojnarski, Michał Jurczyk, Krzysztof Gil

**Affiliations:** Department of Pathophysiology, Faculty of Medicine, Jagiellonian University Medical College, 31-121 Krakow, Poland; ppasieka96@outlook.com (P.P.); patrycja.laczak25@gmail.com (P.Ł.); marcin-wojnarski@wp.pl (M.W.); michal.jurczyk@uj.edu.pl (M.J.); krzysztof.m.gil@uj.edu.pl (K.G.)

**Keywords:** dopamine, dopamine receptors, inflammatory bowel disease, CD, UC, gastrointestinal, gut, animal models, Parkinson’s disease

## Abstract

Background: an increased prevalence of gastro-duodenal ulceration was described almost sixty years ago as prodromal to idiopathic Parkinson’s disease, while duodenal ulcers have been rarely diagnosed in patients with schizophrenia. The cytoprotective role of dopamine in animal models of gastrointestinal ulcerations has also been described. Interestingly, Parkinson’s disease (PD) might share common pathophysiological links with inflammatory bowel disease (IBD) as epidemiological and genetic links already suggest. Thus, the aim of our study was to review the existing literature on the role of the gastrointestinal dopaminergic system in IBD pathogenesis and progression. Methods: a systematic search was conducted according to the PRISMA methodology. Results: twenty-four studies satisfied the predetermined criteria and were included in our qualitative analysis. Due to different observations (cross-sectional studies) as well as experimental setups and applied methodologies (in vivo and in vitro studies) a meta-analysis could not be performed. No ongoing clinical trials with dopaminergic compounds in IBD patients were found. Conclusions: the impairment of the dopaminergic system seems to be a significant, yet underestimated, feature of IBD, and more in-depth observational studies are needed to further support the existing preclinical data.

## 1. Introduction

It is thought that inflammatory bowel disease (IBD) emerged in the Western world with shifts from rural to urban living and is directly associated with the beginning of the industrial revolution in the 18th century, increased air pollution, and dietary changes [1,2]. In fact, a broad spectrum of environmental factors (including breastfeeding, the use of antibiotics in childhood, cigarette smoking, hygiene, and sanitation), microbial dysbiosis, and genetic susceptibility influences the host immune system and can result in mucosal inflammation, characteristic of IBD [3]. An insufficient treatment response stresses the unmet need for a thorough mechanistic understanding of the clinical, genetic, and microbial heterogeneity of IBD [4]. Ulcerative colitis (UC) predominantly affects the rectum and colon, while Crohn’s disease (CD) can affect any segment of the gastrointestinal tract from the mouth to the anus [3]. Interestingly, IBD shares common pathophysiological links with Parkinson’s disease (PD) [5]. Gut inflammation is common in both PD and IBD, and a genetic link between IBD and PD has also been identified [6]. Moreover, a recent systematic review and meta-analysis reported that IBD patients had a 46% increased risk of a PD diagnosis (with a risk ratio of 1.41, and a 95% confidence interval of 1.19–1.66) and this risk remained when patients with CD (28%, with a risk ratio of 1.28, and a 95% confidence interval of 1.08–1.52) and UC (30%, with a risk ratio of 1.30, and a 95% confidence interval of 1.15–1.47) were examined separately [7]. In fact, an increased prevalence of gastro-duodenal ulceration was already described in 1965 as prodromal to idiopathic PD [8], while duodenal ulcers have rarely been diagnosed in patients with schizophrenia [9]. Several reports of the cytoprotective role of dopamine (DA) and dopamine agonists in animal models of gastrointestinal ulcerations have also been published [10,11,12]. DA is indeed an essential modulator of the gut-brain axis [13,14]. 

**Figure 1:**
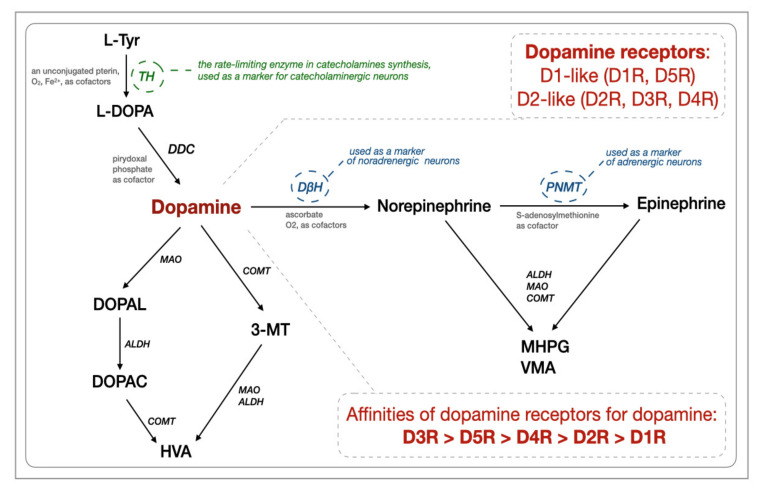
Biosynthesis and degradation of catecholamines together with dopamine receptors (based on [16,23]). The abbreviations are as follows. ALDH: aldehyde dehydrogenases, EC 1.2.1.3; COMT: catechol-O-methyltransferase, E.C. 2.1.1.6; D1R-D5R: dopamine receptors; DβH: dopamine-ß-hydroxylase, dopamine-ß-monooxygenase, E.C. 1.14.17.1; DDC: Dopa decarboxylase, aromatic L-amino acid decarboxylase, E.C. 4.1.1.28; DOPAC: 3,4-dihydroxyphenylacetic acid; DOPAL: 3,4-dihydroxyphenylacetaldehyde; HVA: homovanillic acid; L-DOPA: L-3,4-dihydroxyphenylalanine; MAO: monoamine oxidases, EC 1.4.3.4; L-Tyr: L-tyrosine, 4-hydroxyphenylalanine, a non-essential amino acid; MHPG and PNMT: phenylethanolamine-N-methyltransferase, S-adenosylmethionine, phenylethanolamine N-methyltransferase, E.C. 2.1.1.28; TH: tyrosine hydroxylase, tyrosine 3-monooxygenase, E.C. l.14.16.2; VMA: vanillylmandelic acid; 3-methoxy-4-hydroxyphenylglycol; 3-MT: 3-methoxytyramine, 3-methoxy-4-hydroxyphenethylamine.

A DA molecule consists of a catechol structure (a benzene ring with two hydroxyl side groups) with one amine group attached via an ethyl chain. L-DOPA is a precursor of DA (3,4-dihydroxyphenethylamine) as well as norepinephrine (noradrenaline, NA) and epinephrine (adrenaline). Tyrosine hydroxylase (TH) catalyzes the rate-limiting step in the synthesis of catecholamines (Figure 1), and thus anti-TH antibodies are used as markers of all catecholaminergic neurons. Dopamine-β-hydroxylase (DβH) converts DA into norepinephrine and acts to decrease DA levels. After its release, DA can either be taken up again by the presynaptic terminal (via the dopamine transporter, DAT, and/or by the plasma membrane monoamine transporter, VMAT2) or broken down by enzymes [15]. DA is broken down into inactive metabolites (homovanillic acid, HVA, is the main end-product) by monoamine oxidase (MAO), catechol-O-methyltransferase (COMT), and aldehyde dehydrogenase (ALDH) [16]. The gastrointestinal tract, pancreas, and spleen are the major sources of DA production in the body [17]. In the gastrointestinal tract, DA can be produced by the enteric neurons and non-neuronal tissues/cells such as epithelial and immune cells [17] and gut bacteria [18]. The gastrointestinal dopaminergic system modulates exocrine secretion, fluid absorption, motility, blood flow, and cytoprotection [17,19]. Dopamine-immunoreactive and tyrosine hydroxylase-positive cells were identified throughout the length of the gastrointestinal tract both in humans and rodents. Dopaminergic neurons can be characterized by the presence of TH, DAT, and the absence of DβH [20,21]. Based on rodent studies, all five classes of dopamine receptors (D1-D5R) are present throughout the bowel, with D1, D3, and D5 expressed both in nerve-containing layers and in the mucosa, D4 expressed in the mucosal layer, and D2 expressed in the nerve-containing layers only [22].

A decrease in myenteric dopaminergic neurons was already demonstrated in the colons of Parkinson’s disease patients, which supports the functional importance of dopamine in the human colon. Degenerated dopaminergic neurons in the myenteric plexus might be involved in the pathogenesis of constipation related to PD [24]. Constipation constitutes one of the most common and earliest features of autonomic dysfunction in Parkinson’s disease, even developing fifteen years before the onset of classical motor features. Thus, the early detection of constipation has been proposed as a clinical biomarker of prodromal Parkinson’s disease [25]. The development of constipation is also well recognized in patients with ulcerative colitis, yet the underlying mechanisms remain poorly understood [26]. Therefore, the aim of our study is to systematically review the existing literature on the role of the gastrointestinal dopaminergic system in IBD pathogenesis and/or progression.

## 2. Results

**Figure 2:**
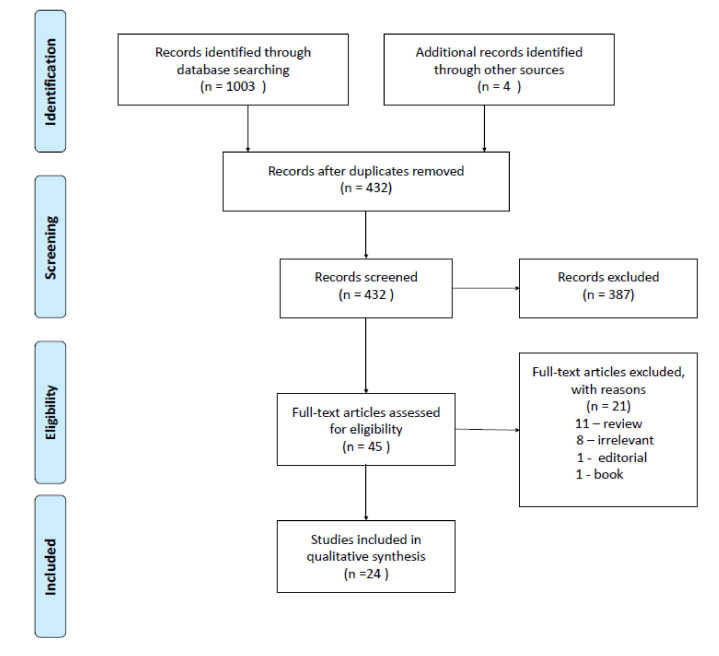
The PRISMA flow diagram. A total of seven exonic single nucleotide polymorphisms (SNPs) were obtained from the DβH gene in the targeted next-generation deep sequencing of IBD patients. One SNP (rs6271) had a significant association in IBD patients when compared to the control group. The allelic frequency distribution of the Arg549Cys polymorphism was significantly different between IBD and control patients. The mean serum DβH concentrations (measured by ELISA) in the IBD cohort ranged from 11.5 ± 5.9 ng/mL (heterozygotes, n = 7), to 124.1 ± 76 ng/mL (wild-type homozygotes, n = 7), to 158.3 ng/mL for the single homozygous SNP. The mean serum DβH concentrations in the control group ranged from 34.4 ± 16.7 ng/mL (heterozygotes, n = 4) and 129.3 ± 16.5 ng/mL (wild-type homozygotes, n = 9). The rs6271 SNP was associated with an increased IBD risk, and there were substantially less DβH proteins in the serum of IBD patients (and controls) with the heterozygous allele for Arg549Cys in DβH [28]. Up-to-date polymorphisms in other enzymes such as phenylethanolamine N-methyltransferase, catechol-O-methyltransferase, or monoamine oxidase were not assessed in IBD patients.

Ultimately, twenty-four studies satisfied the predetermined criteria and were included into this qualitative analysis [27,28,29,30,31,32,33,34,35,36,37,38,39,40,41,42,43,44,45,46,47,48,49,50]. The PRISMA flow diagram is illustrated in Figure 2. No ongoing clinical trials with dopaminergic compounds in IBD patients were found.

### 2.1. In Vivo Results

#### 2.1.1. Human Subjects

All included observational studies and case reports relevant to the research topic are summarized in Table 1.

The following data, though limited, were obtained:

Norepinephrine (NE) tissue levels in both the non-inflamed and inflamed colonic mucosa from CD patients were lower (230 pmol/g and 460 pmol/g, respectively, and estimates were obtained from graphs because no exact values were given in the text) compared to the control patients (~875 pmol/g). L-DOPA (L-3,4-dihydroxyphenylalanine) tissue levels in the inflamed CD mucosa were twice those compared to the non-inflamed mucosa. DA levels in the inflamed mucosa of CD patients were lower (~50 pmol/g) than those of the controls (~135 pmol/g), and DA/L-DOPA tissue ratios, which are a rough measure of aromatic L-amino acid decarboxylase (AADC, also known as DOPA decarboxylase) activity, were reduced only in the inflamed mucosa of CD patients. L-DOPA tissue levels in UC patients were twice as high (~120 pmol/g), both in the inflamed and non-inflamed colonic mucosa, compared with controls (~48 pmol/g). DA levels in the non-inflamed and inflamed mucosa of UC patients were lower (~75 pmol/g and ~50 pmol/g, respectively) than those in the control subjects, and the same was true for DA/L-DOPA tissue ratios. Measurements were performed by high-pressure liquid chromatography (HPLC) with electrochemical detection [31]. However, the patients were not drug naive (see Table 1). Still, in another study, DβH (the enzyme required for the production of NE from DA) expression, assessed immunohistochemically, was up-regulated in lamina propria mononuclear cells (LPMCs) from patients with CD or UC, while a minimal level of DβH was noted in the LPMCs of healthy subjects [29]. Thus, mucosal inflammation should be associated with increased catecholamine synthesis, metabolism, and/or storage. Both neuronal and non-neuronal catecholamine levels were measured in mucosal endoscopic samples [31]. The results obtained by immunohistochemistry implied that LPMCs in particular were an important source of catecholamine synthesis/metabolism during inflammation [29]. To complement the existing data, the mucosal gene expression of all the enzymes involved in catecholamine synthesis and metabolism should be assessed first, preferably both qualitatively (by means of an in situ hybridization) and quantitatively. 

The frequency of the *A1A1* polymorphism (the TaqIA polymorphism of the *D2R* gene, measured by a polymerase chain reaction, which is a restriction fragment length polymorphism) was 3% in controls, 7% in CD, and 7% in UC patients, while the frequency of the *A2A2* polymorphism was 69% in the control population, 67% in CD patients, and 58% in UC patients. Patients with an *A2A2* polymorphism showed a 2.5-times lower risk for the development of refractory CD than *A1A1* and *A1A2* carriers (OR = 0.4, with a 95% confidence interval of 0.21–0.87; *p* = 0.02) [30]. In the gastrointestinal tract, D2 receptors are located post-junctionally and pre-junctionally, and the latter ones exert a negative modulatory effect on the release of acetylcholine from intrinsic cholinergic nerves. The blockage of D2 receptors with antidopaminergics such as domperidone or metoclopramide results in a gastroprokinetic effect [51].

In addition, according to public data sets (GSE47908 and GSE38713), the expression of the dopamine receptor *D5R* gene (measured by RT-qPCR) was significantly decreased in the colons of active as well as in-remission UC patients [38]. The expression of heteromeric complexes formed by D5R and CCR9 (the C-C chemokine receptor type 9) were up-regulated (assessed immunohistochemically via lymphoid cells) in the intestinal mucosa of IBD patients compared to healthy controls [27]. The involvement of D5R in the control of gut homeostasis in humans remains poorly explored, it might be responsible, together with D1R, for the regulation of the mucosal barrier integrity [52,53]. Interestingly, a range of physiologically relevant dopamine signaling is expanded by the ability of dopamine receptors to interact with other dopamine receptors and with receptors of other endogenous signaling ligands [54]. The CCR9:D5R heteroreceptor is such an example, and its presence inevitably adds another layer of complexity to the control of mucosal inflammation. These results are especially interesting due to the fact that high tissue levels of DA can activate low-affinity receptors to promote immunosuppression, whereas low levels of DA, such as those seen in inflammation, can activate high-affinity pro-inflammatory receptors, such as D5R (Figure 1) [55].

All of the above-mentioned studies clearly imply the importance of catecholaminergic neurotransmission during the intestinal inflammation that is characteristic of IBD. Case studies (Table 1 [32,33,34,35,36,37]) support this importance yet their clinical significance is insufficient. All in all, the human subject data is preliminary due to the limited number of human subjects included in the studies and the incomplete picture of the role of gastrointestinal DA in the development and progression of IBD pathology.

#### 2.1.2. Animal Models

A wide range of IBD-specific animal models were analyzed from the articles chosen for our review, such as the chemical colitogens 1-fluoro-2,4-dinitrobenzene (DNFB), dextran sulphate sodium (DSS), idoacetamide (IA), and 2,4,6-trinitrobenzene sulphonic (TNBS) acid, as well as biological models including the adoptive T-cell transfer in genetically modified mice that were interleukin-10 (IL-10)-deficient and were homozygous T-bet−/− Rag2−/− (also known as TRUC). 

Because of their simplicity, chemical colitogens can be used in several experimental protocols. They induce intestinal inflammation by the disruption of the mucosal barrier and/or by triggering hapten-associated hypersensitivity reactions, yet they do not recapitulate the complete IBD pathology [56]. Perhaps the most widely used DSS-induced experimental colitis is characterized by the death of epithelial cells, intestinal barrier dysfunction, and subsequent inflammation due to the dissemination of pro-inflammatory intestinal contents (e.g., bacteria and their products) into underlying tissue. Acute and chronic forms of the pathology can be induced in transgenic and immunodeficient animals as well. It most closely resembles human UC; however, unlike in the human disease, T and B cells were not required for the development of DSS-induced colitis [57,58]. On the other hand, TNBS-induced colitis (the Th1-mediated immune responses) mimics CD more closely [59].

Dysregulated adaptive immunity is not the only necessary requirement for the development of human IBD, which limits the use of the adoptive T-cell transfer mouse model of colitis. The adoptive transfer of CD4 + CD45RBhigh T-cells (naive T-cells, depleted of the Treg cell population) from healthy wild-type (WT) mice into recipients that lack T- and B-cells induces pancolitis and small bowel inflammation following T-cell transfer, which thus successfully recapitulates the intestinal inflammation observed in human UC and CD [60]. 

Among a variety of genetic models, the interleukin-10(IL-10)-deficient mice model is characterized by a range of intestinal alterations, including extensive mucosal hyperplasia, inflammatory reactions, and the aberrant expression of major histocompatibility complex class II molecules on epithelial cells. Furthermore, mutants kept under specific pathogen-free conditions develop only a local inflammation limited to the proximal colon [61]. Conversely, TRUC mice develop spontaneous juvenile ulcerative colitis resulting from a pro-inflammatory response to the commensal microbiota (germ-free TRUC mice do not develop the disease) driven by dendritic cells and TNFα. When co-housed with WT mice, this mouse strain can induce colitis in the normal hosts via the transmission of the colitogenic intestinal microbiota [62].

Additionally, selected studies with oxidopamine, also known as 6-hydroxydopamine (6-OHDA; 2,4,5-trihydroxyphenethylamine, a neurotoxin capable of destroying noradrenergic and dopaminergic neurons) as well as MPTP (1-methyl-4-phenyl-1,2,3,6-tetrahydropyridine, a prodrug to the dopaminergic neurotoxin MPP+) were evaluated due to their relevance and applicability to our selected topic. All relevant details, including pharmacological interventions, are summarized in Table 2. The results of the selected studies in relation to the peripheral, especially gastrointestinal, dopaminergic system are extensively reported below.

##### 2.1.2.1. Gut Dopamine Levels in IBD-Specific Animal Models

The inflamed colonic mucosa levels (measured by HPLC with electrochemical detection) of DA and L-DOPA were lowered (by around 35% and 60%, respectively) in TNBS-treated compared to saline-treated rats, while ileal levels of L-DOPA, DA, and DOPAC were comparable in saline-, ethanol- and TNBS-treated rats. The levels of L-DOPA, DA, and DOPAC in saline-treated (but not ethanol-treated) rats were similar in the ileal and colonic mucosae [46]. Colonic DA levels (measured by HPLC) were reduced (by around 33.3%) after DSS treatment in mice [38]. These results reflect DA depletion observed in human samples and suggest that colonic inflammation and a breach in the integrity of the mucosal barrier observed in TNBS- and DSS-induced colitis is associated with catecholaminergic dysregulation.

DA levels (measured by ELISA) in stool samples were decreased (by around 66%) in untreated TRUC mice, and vancomycin (an antibiotic used to treat life-threatening infections by Gram-positive bacteria) was used to treat the active colitis in combination with gentamicin (an antibiotic used to treat a wide range of bacterial infections, especially by Gram-negative bacteria) induced remission. There was also a trend toward lower levels of DA in vancomycin-treated versus untreated mice [44]. The TRUC mouse model can be used to assess the host-commensal relations at the mucosal surface, and *K. pneumoniae* has already been implicated in the TRUC pathogenesis [63]. Since members of the Klebsiella genus share the genomic potential to fully metabolize catecholamines, active colitis in this model, along with its Enterobacteriaceae enrichment, was expected to be associated with decreased levels of fecal DA. This study further confirms the ability of intestinal microflora to modulate the gastrointestinal dopaminergic system [44]. Although vancomycin has been an important medication in the treatment of IBD flares associated with *Clostridium difficile*, as well as dysbiosis, and should also be considered a frontline antibiotic therapy in IBD [64], its use might be associated with alterations in the gastrointestinal catecholamine turnover and the expansion of colitogenic bacteria (Mucispirillum, Desulfovibrio, and Helicobacteraceae) [44]. Yet, no comparison was made between TRUC mice treated with antibiotics versus wild-type mice.

##### 2.1.2.2. Colonic TH, AADC, and DAT Expression in IBD-Specific Animal Models

TH protein levels that are assessed by means of immunohistochemistry were induced in the colonic mucosa after TNBS and DSS treatment in comparison with control mice. TNBS- or DSS-induced TH immunoreactivity was detectable in the cytoplasm of lamina propria mononuclear cells [45]. The activity (measured by HPLC with electrochemical detection) of AADC (also known as DOPA decarboxylase), with L-DOPA (0.1–10 mm) used as a substrate, was higher in homogenates of the ileal (but not colonic) mucosae from TNBS-treated rats versus controls [46]. Since TH catalyzes the rate-limiting step in this synthesis of catecholamines, these results further support the observation that catecholaminergic dysregulation is characteristic for the colonic inflammation observed in TNBS- and DSS-induced colitis. Although AADC is not the rate-limiting step in DA synthesis, its different expression levels in small and large intestinal mucosae should be further investigated. Conversely, colonic mucosal TH levels (measured by Western blot) in rats were lower after an IA enema versus the control group. Chronic colitis in IL-10 KO mice was associated with a half-fold decrease in TH levels versus the wild-type littermates [43]. Furthermore, TH protein levels were decreased in the colon, but remained unchanged in the brain tissue of MPTP-treated rats [49]. However, no data regarding the TH protein levels in animals treated with both MPTP and IA was available.

DAT-positive staining in the normal colon was expressed in epithelial, endothelial, and neural cells of both enteric plexuses, while IA-damaged colonic mucosae exhibited decreased DAT-immunoreactivity. In inflamed colonic mucosae, DAT-positive staining was predominantly localized on the surface of colonocytes [33]. DAT removes DA from the synaptic cleft and deposits it into surrounding cells, which terminates the signal of the neurotransmitter [65]. Thus, its decreased availability, as observed in IA-damaged colonic mucosae, should influence the turnover of catecholamines in colonocytes, and could further impact the balance of neurotransmitters throughout the intestinal wall [66].

With regard to the analysis of these results, it should also be stressed that quantitative methods such as HPLC and Western blot differ in terms of precision and reproducibility, and when compared with qualitative methods such as immunohistochemistry, the difference in protein expression in neuronal and non-neuronal cells cannot be determined. 

##### 2.1.2.3. The Colonic Expression of Dopamine Receptors in IBD-Specific Animal Models

Protein levels of the dopamine receptor D2R in the colonic mucosa of IA-treated rats and IL-10 knock-out mice were increased threefold in comparison to normal wild-type controls [43]. 

Gene expression levels of the dopamine receptors *D1R*, *D4R*, and *D5R* were highly expressed in CD45+ lamina propria (LP) hematopoietic cells, while the expression of *D2R* and *D3R* was relatively low in both colonic epithelial and LP immune cells of wild-type mice. A high (four times higher, in fact, than in the other immune cells) expression of D5R in macrophages was also observed. CX3CR1+ macrophages were adjacent to TH positive neurons in the LP layer. A wide distribution of D5R signaling in F4/80+, Arg1+ M2, and Inos+ M1 macrophages in the LP was observed immunohistochemically [38]. These data suggest that D5R-mediated signaling regulates colonic macrophages. Since TH positive neurons could be of either extrinsic of intrinsic origin, the intestinal neuron-macrophage crosstalk should be further explored.

The disruption of the mucosal heteromeric complexes formed by D5R and CCR9 was observed to impair the entrance of CD4+ T-cells into the colonic and cecal mucosae in DSS-treated WT congenic mice, which received a transfer of CD4+ T-cells pretreated with transmembrane peptides (peptides mimicking the transmembrane domains from CCR9 and D5R that are involved in the interacting interference). Those peptides were designed to disrupt the complexes, and the transmembrane regions were predicted by 3-D modeling following the criteria used in the crystallization of G protein-coupled receptors. Collectively, these results and the ones from human subjects suggest that the D5R and CCR9 heteromers drive lymphocyte infiltration into the colonic lamina propria upon gut inflammation, which may have therapeutic implications in IBD [27].

##### 2.1.2.4. Immune Alterations Associated with D3R Deficiency

D3R-deficient mice were unresponsive to DSS-induced inflammatory colitis as evidenced by the disease activity index (DAI, which was close to zero) and histological analyses when compared with D3R-sufficient mice. *Drd3−/−* mice were resistant to DSS-induced inflammatory colitis when previously co-housed with *Drd3+/+,* and *Drd3+/+* mice were susceptible to DSS-induced inflammatory colitis even when previously co-housed with *Drd3−/−* mice [39]. Thus, co-housing experiments, which enabled the transfer of gut microbiota, ruled out the role of microbiota in the phenotype presentation of the D3R-deficient mice. 

The D3R deficiency in mice also led to the enhanced recruitment of Treg cells in the gut mucosa upon DSS treatment, especially into the colonic lamina propria. DSS-treated mice receiving a suboptimal amount (meaning lower, i.e., 3 × 10^5^ cells per mouse when compared with levels that were previously established as optimal) of D3R-deficient Treg cells presented with an improvement of the colitis manifestation in comparison with DSS-treated mice receiving D3R-sufficient *GFP+CD4+* Treg cells, or those that were only DSS-treated (without the Treg cell transfer). Wild-type DSS-treated mice receiving D3R-deficient Treg cells displayed a significant reduction in body weight loss and a reduction in their histopathological score (a half-fold decrease) as well as showing a half-fold reduction in the Th17 frequency in mesenteric lymph nodes. Moreover, the transfer of ex vivo RV-shDrd3-transduced Treg cells at the beginning of DSS-induced colitis into WT (*Cd45.2+/+*) mice reduced the disease manifestation when compared with the transfer of RV-control-transduced Treg cells. DSS-treated mice that received the i.v. transfer after the beginning of the DSS treatment also had reduced disease severity. A one-fold increase of inflammatory infiltration into the colon was observed for RV-shDrd3-transduced Treg cells in comparison with the control-transduced Treg cells [39]. Thus, D3R signaling is a critical regulator of Treg cell migration into the intestinal mucosa upon gut inflammation.

##### 2.1.2.5. Immune Alterations Associated with D5R Deficiency

No differences in the weight or histopathology of colons between *Drd5−/−* or WT mice were observed before the DSS treatment, yet more severe colitis (greater body weight loss, a higher DAI score, and shorter colons) was observed in *Drd5−/−* mice after the DSS administration. A D5R deficiency increased inflammatory cell infiltration with greater damage of the mucosal epithelium. The serum levels (measured by ELISA) of TNFα, IL-6, and CCL2 (also known as MCP-1, monocyte chemoattractant protein-1) were significantly increased in the *Drd5−/−* group compared with the WT group. A D5R deficiency was also associated with increased numbers of Inos*+* M1 cells but reduced numbers of *Arg1+* M2 cells in the colon compared with the WT mice. The WT mice that were reconstituted with *Drd5*−/− bone marrow were more prone to DSS-induced colitis with greater body weight loss, a higher DAI score, shorter colons, and more infiltrating inflammatory cells as well as higher levels of TNFα, IL-6, and CCL2 in serum. Mice that received *Drd5−/−* CD45.2/WT CD45.1 bone marrow cells had a higher M1 polarization and a lower M2 polarization than the WT donors after DSS-induced colitis. However, the *Drd5−/−* or WT recipient mice reconstituted with bone marrow cells that were isolated from the WT mice demonstrated comparable body weight loss, DAI scores, colon length, histopathology, and serum inflammatory cytokines [38]. Accordingly, the M1/M2 macrophage polarization upon gut inflammation is dependent on D5R signaling.

Furthermore, co-housing experiments revealed that *Drd5−/−* single-housed mice and co-housed mice (housed with WT mice) still had a higher presence of colitis-associated microbiota (assessed by 16s rRNA sequencing), such as Prevotellaceae and Clostridia_UCG-014, and lower abundance of protective bacteria, including Bacteroidaceae and Tannerellaceae. The microbiota transfer due to co-housing was not protective against the severe manifestation of subsequently DSS-induced colitis in *Drd5−/−* mice [38]. Thus, D5R-signaling (but not D3R [39]) should control the proliferation of (pathological) microflora.

##### 2.1.2.6. *Rag1−/−* Mice (Lacking T- and B-Lymphocytes) and DR Deficiencies

The transfer of normal naïve CD4+ T-cells induced a weight loss in recipient *Rag1−/−* mice, while *Drd3−/−* naïve CD4+ T-cells caused a smaller weight loss [40]. Similarly, *Rag1−/−* mice receiving D3R-deficient Treg cells displayed a smaller body weight loss in comparison with mice receiving D3R-sufficient Treg cells [39]. Histological analyses revealed that Drd3−/− naïve CD4+ T-cells induced mild mucosal inflammation compared to normal naïve CD4+ T-cell recipients. The proportion of IFNγ+ cells was reduced in recipients of Drd3−/− naïve CD4+ T-cells when compared with the recipients of normal naïve CD4+ T-cells; yet, recipients of D3R-deficient naïve CD4+ T-cells showed similar frequencies of IL-17A+ and IL-17A+IFNγ+ CD4+ T-cells compared to those of the recipients of WT CD45RB CD4 T-cells. The percentage of Foxp3-expressing cells was reduced in recipients of *Drd3−/−* naïve CD4+ T-cells [40]. Furthermore, *Rag1−/−Drd3−/−* as well as *Rag1−/−Drd3+/+* mice were susceptible to DSS-induced inflammatory colitis, and both genotypes presented with similar body weight loss [39]. Collectively, D3R-signalling is a major regulator of adaptive immunity (especially in Treg cells) favoring the development of experimental colitis.

*Rag–/–* mice receiving D5R-deficient naïve CD4+ T-cells manifested less severe colitis in comparison with mice receiving D5R-sufficient naïve CD4+ T-cells, and their body weight increased with the time course of the experiment, similar to the expected body weight increase with age in healthy mice. The D5R deficiency in CD4+ T-cells was also associated with a reduced CD4+ T-cell infiltration into the colonic mucosa, but without affecting T-cell differentiation, survival, or proliferation. However, the D5R deficiency and CCR9 deficiency in CD4+ T-cells reduced the development of colitis in a similar manner. The arrival of D5R-deficient CD4+ T-cells in the colonic LP and the mesenteric lymph nodes was impaired compared with D5R-sufficient lymphocytes, and D5R signaling promoted the selective migration of CD4+ T-cells into the gut-associated tissues. At the same time, the CCR9 expression was selectively increased in frequency and density on CD4+ T-cells infiltrating the colonic LP only. Furthermore, abundant staining (positively stained cells compared to the total number of cells with leukocyte sizes) for CCR9:D5R heteromeric complexes was present in colonic samples from Rag1–/– mice that were recipients of D5R-sufficient CD4+ T-cells, and in the samples obtained from recipients with a mixture of D5R-sufficient and D5R-deficient naïve CD4+ T-cells (yet the number of positive cells was around six times lower), while the staining was negligible in samples obtained from recipients of D5R-deficient naïve CD4+ T-cells [27]. Thus, a D5R deficiency in CD4+ T-cells limits the development of experimental colitis and impairs its migration into the gut mucosa. What is more, CCR9:D5R heteromeric complexes can be formed in CD4+ T-cells.

##### 2.1.2.7. *Rag1−/−* Mice (Lacking T- and B-Lymphocytes) Together with 6-OHDA Treatment (Unselective Sympathetic Dysfunction) or Surgical Sympathectomy

Intraperitoneal treatment with 6-OHDA (chemical sympathectomy) produced no differences in body weight loss, colon weight, or colonic cytokine levels over time between *Rag1−/−* mice, but produced histological features characteristic of colitis, especially a decrease in the number of goblet cells. *Rag1−/−* mice after the sympathectomy of the superior mesenteric nerve (an intestine-specific sympathectomy) did not gain weight over a time period after the surgery. Colon weights were higher, and the ileal NE levels were lower (a 5.7-fold decrease) in the sympathectomy group compared to the sham control group. The total histology scores for colitis, as well as the colonic mRNA expressions of *IL-1β*, *IL-6*, and *IL-10* were higher after the sympathectomy, compared to the sham. No differences in the total innate immune compartments in the mucosa were observed between the groups, yet an elevated frequency of the absolute cell number of immature monocytes after the sympathectomy was observed [48]. These results support the importance of the sympathetic innervation in gastrointestinal immune regulation. Since either *Rag1−/−* mice or wild-type mice, upon the sympathectomy, did not develop colitis [48], monocyte and macrophage cell populations should be key players in the observed experimental colitis. 

##### 2.1.2.8. Colonic and Immune Alterations in Models Primarily Associated with Chemical Sympathetic Denervation Together with Disease-Specific Models

A pharmacological 6-OHDA-induced sympathectomy attenuated TNBS-induced colitis in rats, as indicated primarily by macroscopic and histological scores. The protective role of lidocaine in TNBS-induced colitis was demonstrated, and the effect was enhanced in the absence of sympathetic nerves. Thus, the removal of the pro-inflammatory role of sympathetic nerves by 6-OHDA, and the administration and inhibition of the release of transmitters from enteric nerves by lidocaine in the presence of primary afferents, resulted in the greatest reduction of colitis manifestation (evidenced by macroscopic and microscopic scoring and myeloperoxidase activity) [50].

The myeloperoxidase (MPO) levels (considered as a marker of neutrophil activation and degranulation) in colonic mucosae did not differ between MPTP- and saline-treated rats with IA-induced colitis. In peripheral blood, IA-induced colitis in both MPTP- and saline-treated rats was associated with an increase in neutrophils and granulocytes in comparison to animals without colitis. Decreased ROS production by monocytes and an increased number of CD69-positive monocytes were observed in MPTP-treated rats compared to saline-treated rats during colitis. The CD14 surface expression was increased in MPTP-treated rats without colitis, but it decreased in both saline- and MPTP-treated rats with colitis [49].

##### 2.1.2.9. Dopamine Agonists and Experimental Colitis

Bromocriptine (primarily a D2R subfamily agonist, with a higher affinity to D2/3R, see Table 3) administration, at the dose of 10 mg/kg via i.p. for 5 days after challenge enemas, reduced mortality rate among DNFB-treated mice (3%) compared to the control (6%) group, and reduced the morphological signs of colitis. Reduced ulcerations, necroses, and lower edema scores of the colonic LP and submucosa, as well as less pronounced inflammatory cell infiltration was observed in bromocriptine DNFB-treated mice compared with the control [47].

Quinpirole (D3/4R agonist, see Table 3) administration, at the dose of 10 mg/kg via i.g. for 5 days starting from the second day of the experiment, reduced DAI in rats with IA-induced colitis. Extensive ulceration, as well as acute and chronic inflammatory cells’ infiltration with no or minimal mucosal regeneration was characteristic for saline- compared to quinpirole-treated rats. Quinpirole reduced the size of colonic lesions, colonic dilatation, and the ratio of colon wet weight/100 g of body weight in rats with IA-induced colitis. Pretreatment with quinpirole 30 min before the IA enema reduced Evans blue extravasations in the colonic mucosa, which was correlated with a decreased phosphorylation of c-Src and Akt signaling molecules. However, quinpirole treatment did not affect an IA-induced increase in colonic epithelial permeability [43]. 

Quinpirole administration, at the dose of 10 mg/kg via i.g. for 13 days, decreased the colon wet weight in IL-10 KO mice (compared to the saline-treated mice). There were no apparent signs of diarrhea nor any significant changes in body weight in IL-10 KO mice treated with saline or quinpirole. The spleen weight and MPO activity was reduced 1.5-fold and 1.3-fold, respectively, in quinpirole-treated IL-10 KO mice. Histological analyses demonstrated no inflammatory difference in saline- vs. quinpirole-treated IL-10 KO mice [43].

Cabergoline (a long-acting D2R agonist, see Table 3) administration, at the dose of 10 µg/kg via i.g., had no influence on clinical and macroscopic signs of IA-induced colitis in the rats or in vascular permeability. However, the dose of 50 µg/kg improved the DAI score, the ratio of colon wet weight/100 g of body weight, and morphological signs including colonic lesions and dilatation, as well as increasing the survival rate in the IA-induced model of colitis [43]. 

Furthermore, cabergoline (20 uM) administration to the larval media for the duration of TNBS exposure suppressed TNBS-induced nitric oxide production in the cleithrum and notochord at 6 days post-fertilization (dpf) of the larvae of zebrafish (*Danio rerio*). An addition of cabergoline (20 uM) at 5 dpf suppressed the existing TNBS-induced nitric oxide production to baseline levels. Moreover, cabergoline reduced post-infection embryo survival. DSS- and TNBS-induced neutrophilic inflammation was also silenced in zebrafish larvae treated with cabergoline at all examined doses (5 to 50 uM). Zebrafish models, including those with chemically induced entero-colitis, are not directly transferable to rodent or human studies; however, their relevance to IBD has already been reported, and could provide a low-cost animal model of acute colitis for the initial stages of anti-inflammatory drug discovery programs [41].

SKF-38393 (a D1R subfamily agonist with a higher affinity to D5R, see Table 3) administration, at the dose of 10 mg/kg via i.p., reduced the clinical signs of DSS-induced colitis in WT mice, observed as reduced weight loss, a lower DAI score, reduced shortening of the colon length, less histopathological findings, and lower serum levels of the cytokines TNFα, IL-6, and CCL2, whereas those effects were impaired in *Drd5−/−* mice [38].

Thus, regardless of the experimental model, dopamine agonists reduced morphological signs of colitis.

##### 2.1.2.10. Dopamine Antagonists and Experimental Colitis

Domperidone (a D2/D3R peripheral-specific antagonist, see Table 3) administration, at the dose of 5 mg/kg via i.p. for 5 days after the challenge enemas, increased mortality rate among DNFB-treated mice (20%) compared to the control (6%) group. More pronounced ulcerations, necroses, inflammatory cell infiltration, and extensive hemorrhages were observed in domperidone DNFB-treated mice compared to control mice [47].

Berberine (a D1/D2R antagonist, see Table 3) administration at 400 μL/mL in double-distilled drinking water reduced weight change, inhibited colon shrinkage, reduced the levels of intestinal damage, and infiltrated lymphocytes in DDS-induced colitis [42].

Haloperidol (a D2R subfamily antagonist, see Table 3) administration at 7.5 uM to the larval media, together with 0.25% or 0.5% DSS, increased neutrophilic inflammation in the intestine and trunk of zebrafish. However, haloperidol treatment did not affect neutrophilic inflammation or survival following an exposure to TNBS. It should be noted that the titrations of haloperidol at doses above 10 uM revealed severe developmental defects [41].

##### 2.1.2.11. Adrenergic Compounds and Experimental Colitis

RX821002 (an α2-adrenoceptor antagonist, see Table 3) administration, at the dose of 10 mg/kg via i.p. 2 h after TNBS colitis induction which was repeated daily resulted in a survival rate of 84.6% (compared to 70.6% in TNBS-treated group only) on day 7, as well as improved histological signs with a reduction in inflammatory activity, neutrophil infiltration, and MPO activity. It also reduced the production of inflammatory cytokines in colonic homogenates, such as TNFα and IL-1β, when compared with the control. RX821002 also resulted in an amelioration of DSS-induced colitis, as shown by a decrease in DAI, an improvement in stool consistency, reduced rectal bleeding, reduced colonic MPO activity, reduced inflammatory activity, and immune cell infiltration compared with mice treated with DSS only or UK14304-treated mice. Moreover, RX821002 reduced the production of inflammatory cytokines in colonic homogenates, such as TNFα and IL-1β, when compared with DSS-induced colitis [45].

UK14304 (an α2-adrenoceptor agonist, see Table 3) administration, at the dose of 2 mg/kg via i.p. 2 h after TNBS colitis induction, which was repeated daily, resulted in the lowest survival rate of 57.9%, and intensive body weight loss. The highest histological score, with severe mucosal tissue damage, massive immune cell infiltration, and the highest MPO activity with elevated levels of TNFα together with IL-1β, was observed among UK14304- and TNBS-treated mice. UK14304 administration worsened body weight loss, increased DAI, and increased MPO activity in DSS-treated mice. Histological examinations of the distal colon of UK14304-treated mice with DSS-induced colitis showed severe mucosal tissue damage, multi-focal dropouts of crypts, and an infiltration of inflammatory cells, such as macrophages, lymphocytes, and neutrophils. UK14304 administration elevated the production of TNFα and IL-1β in colonic homogenates when compared with RX821002-treated mice [45]. Collectively, α2-antagonists, due to their anti-inflammatory properties, could limit the experimental progression. 

### 2.2. In Vitro/Ex Vivo Results

#### 2.2.1. Jurak Cells (the Human T Lymphocyte Cell Line) and the Presence of CCR9:D5R Heteromers

The bioluminescence resonance energy transfer assay in co-transfected Jurkat cells revealed a close proximity (10–100 Å) between CCR9 and D5R, which proves a direct interaction between these receptors. SCH23390 (a D5R antagonist) was able to attenuate the increased ERK1/2 phosphorylation induced by the single stimulation of either D5R or CCR9, and a cross-antagonism in cAMP assays was also observed. When Jurkat cells were preincubated with transmembrane peptides that disrupted the CCR9:D5R heteromeric complex assembly (TM5C, TM6C, TM5D, and TM6D), ERK1/2 phosphorylation and cAMP accumulation were abolished [27]. These results considerably support the already discussed in vivo observations. 

#### 2.2.2. The Influence of D3R Deficiency on T-Cells

The proliferative ability of CD4+ T-cells derived from *Drd3−/−* mice did not differ from those of *Drd3+/+* mice. Drd3−/− CD4+ T-cells induced IL-17A expression, similar to the *Drd3+/+* CD4+ T-cells under Th17-skewing conditions, but showed a lower frequency of IFNγ–producing cells when exposed to Th1-polarizing signals. Among IFNγ+ cells, CD4+ T-cells lacking D3R expressed lower amounts of IFNγ on a per cell basis. Additionally, silenced D3R expression on activated WT CD4+ T-cells resulted in a reduced frequency of IFNγ–producing cells when compared with CD4+ T-cells transduced with a non-silencing control retrovirus. WT CD4+ T-cells activated under Th2-skewing conditions expressed marginal levels of Th2-related cytokines, while D3R-deficient CD4+ T-cells produced significantly more IL-13. *Drd3−/−* CD4+ T-cells cultured under Th2-polarizing conditions resulted in more Gata3 and IL-4 production in comparison with WT CD4+ T-cells [40]. Thus, D3R-signaling favors the inflammatory potential of CD4+ T-cells.

A D3R deficiency in total CD4+ T-cells, which also includes the subset of Treg cells, resulted in impaired Th1- and Th17-mediated immunity in the antigen-specific immunization model induced by the ovalbumin-derived peptide OT-II (OVA323-339). Both the frequency of IL-10-producing Treg cells isolated from mesenteric lymph nodes and the colonic lamina propria, as well as the intensity of IL-10 production, was higher in D3R-deficient Treg cells in comparison with D3R-sufficient Treg cells following DSS treatment. However, a D3R deficiency did not increase the production of TGFβ or the surface expression of CTLA-4, CD39, or CD73 in Treg cells from mesenteric lymph nodes and the colonic lamina propria following DSS treatment [39]. Accordingly, D3R signaling reduces the suppressive activity and IL-10 production in Treg cells.

What is more, a D3R deficiency resulted in a selective increase in the frequency and density of the CCR9 expression in Treg cells (CD4+ GFP+). A higher CCR9 expression in D3R-deficient Treg cells from the mesenteric lymph nodes of mice with chronic colitis was also observed. A D3R deficiency induced a lower α4β7 integrin (responsible for T-cell homing into gut-associated lymphoid tissues) expression in colonic Treg cells following DSS treatment [39]. 

#### 2.2.3. The Influence of Dopamine on Bone Marrow-Derived Macrophages (BMDMs)

The DA treatment inhibited LPS (100 ng/mL)- and INF-γ (50 ng/mL)-induced expressions of M1-associated genes in bone marrow-derived macrophages (BMDMs), including Inos, Tnf, and Il6, as well as decreasing the protein levels of iNOS, TNFα, and IL-6. The DA (0.5 μM) co-treatment with MAO and COMT inhibitors improved its inhibitory effect. DA (20 μM) reduced the expression of the CD86 marker on macrophages under M1 conditions. The inhibitory effect of DA on the mRNA expression levels of M1-associated genes, including Inos, Tnf, and Il6 in response to LPS/IFNγ was lost, while the inhibitory effects of DA on TNFα and IL-6 protein levels in response to LPS/IFNγ stimulation were impaired in *Drd5−/−* cells. The inhibitory effect of DA on iNOS protein levels in macrophages was blocked in D5R deficiency. *Drd5−/−* cells were resistant to the inhibitory effects of DA on CD86 expression under M1 conditions. Additionally, DA augmented the expression of M2-associated genes (*Arg1*, *Mrc1,* and *Ym1)* in response to IL-4 (10 ng/mL) and IL-13 (10 ng/mL) treatments. DA may also increase the induction of CD206+ M2 macrophages in response to IL-4/IL-13, but failed to increase the expression of M2-associated genes (*Arg1*, *Mrc1*, and *Ym1*) in *Drd5−/−* cells [38].

DA-treated M1 WT BMDMs had a characteristic architecture in comparison with the M1 WT, M1 *Drd5−/−*, and DA-treated M1 *Drd5−/−* cells. TNFA_SIGNALING_VIA_NFKB (a pro-inflammatory pathway) was down-regulated in DA-treated M1 WT BMDMs compared with untreated M1 WT cells. The CREB_PATHWAY (promotes anti-inflammatory immune responses, for example, via the inhibition of NF-κB activity) was up-regulated in M2 WT BMDMs after DA treatment, and the CREB_PATHWAY was also the leading gene set in M2 WT BMDMs relative to *Drd5−/−* cells in the presence of DA. Consistently, DA increased the phosphorylation of CREB WT BMDMs after IL-4/IL-13 stimulation, but such effects of DA were impaired in *Drd5−/−* BMDMs. Moreover, an increase in the expression of a variety of genes associated with M2 macrophage and CREB signaling, such as *Arg1*, *Chil3*, *Mrc1*, *Socs1*, and *Il10* in WT but not *Drd5−/−* BMDMs under M2 conditions in the presence of DA was observed [38].

Clearly, dopamine via D5R-signalling can inhibit M1 polarization and promote M2 polarization through the activation of the CREB pathway. 

#### 2.2.4. The Influence of Berberine on Mesenteric Lymph Node (MLN) Lymphocytes 

MLN lymphocytes from DSS-treated mice secreted IFNγ and IL-17 (Table 4) following the stimulation of anti-CD3 and anti-CD28 antibodies, yet the levels were decreased by the administration of berberine. The secretion of IFNγ, TNFα, IL-6, and IL-1β from LPS-stimulated MLN lymphocytes was inhibited by berberine (1 μM and 10 μM), and the efficacy of berberine for inhibiting innate immune responses was similar to that of SCH23390 (a selective dopamine D1R-subfamily antagonist) and L750667 (a selective dopamine D2R-subfamily antagonist, especially D4R) [42]. 

#### 2.2.5. The Influence of Adrenergic Compounds on Lamina Propria Mononuclear Cells (LPMCs)

RX821002 (10 nM) administration down-regulated the production of TNFα and IL-1β, whereas UK14304 (5 nM) administration induced the secretion of the pro-inflammatory cytokines from LPMCs that were isolated from TNBS-treated mice. LPS (50 ng/mL) induced a pro-inflammatory cytokine release from LPMCs, but pretreatment with RX821002 (2 nM, 10 nM) decreased TNFα and IL-1β production. Conversely, UK14304 enhanced the pro-inflammatory cytokine secretion from LPMCs induced by LPS. The pro-inflammatory cytokine production of LPMCs treated with LPS (50 ng/mL) and RX821002 (10 nM) was lower than the cells treated with LPS (50 ng/mL) and UK14304 (1 nM or 5 nM) [45]. These results support the in vivo observations.

## 3. Discussion

This review acknowledged the vital role of the peripheral dopaminergic system in the development and progression of inflammatory bowel disease, due to its both pro-inflammatory and anti-inflammatory activities. Its role in T-cell driven inflammation is especially highlighted by both ex vivo and in vivo studies [27,39,40,42,47]. Unfortunately, the small amount of research involving human subjects was limited to observational studies, with no interventional ones. Above all, a decrease in the DA/L-DOPA (together with 5-HT/L-5HTP) tissue ratio in CD and UC patients was marked, which implied that the decarboxylation of L-DOPA (and L-5-hydroxytryptophan) might be compromised in IBD patients [31]. Similarly, decreased levels of DA and L-DOPA in the colonic mucosa in TNBS-treated rats [46], DA levels in the colonic mucosa in DSS-treated mice [38], and fecal DA levels in active colitis in TRUC mice [44] were observed. Additionally, DAT immunoreactivity in IA-damaged colonic mucosae was decreased [43]. The reported TH expression levels in colonic mucosae among TNBS-, DSS-, and IA-treated rodents as well as IL-10 mice were inconsistent [43,45]. Physiologically, human mesenteric organs, including the gastrointestinal tract, spleen, and pancreas, are responsible for the production of substantial amounts of DA. Gastrointestinal DA is definitely much more than just a metabolic intermediate in the formation of NE and epinephrine, as it modulates jejunal sodium absorption and mucosal blood flow, inhibits gut motility, and stimulates exocrine secretions [17]. Nevertheless, the expression of the rate-limiting enzyme for DA synthesis, or DAT, throughout human gastrointestinal IBD samples has not been reported.

Gastrointestinal DA is produced and released not only by enteric neurons but also by different immune cells, including macrophages, dendritic cells, and lymphocytes, as was demonstrated by Eisenhofer et al. based on the cellular distribution of TH throughout the healthy human gastrointestinal tract [17]. Gastrointestinal DA is also a fundamental mediator between the nervous and immune systems. Moreover, a fraction of immune cells in the LP of stomach and duodenum exclusively express mRNA for DRs and TH, but not for DβH or phenylethanolamine N-methyltransferase [67]. DA exerts its effects through five receptors (D1RD1–D5R) belonging to the G protein-coupled receptor (GPCR) superfamily. Two D1-like receptor subtypes (D1R and D5R) couple to the G protein Gs and activate adenyl cyclase. The other receptor subtypes that belong to the D2-like subfamily (D2R, D3R, D4R) inhibit adenyl cyclase and activate K+ channels [68]. Both innate and adaptive immunities can be altered by dopaminergic deregulation, and consequently should contribute to the development of numerous inflammatory diseases including IBD and Parkinson’s disease, as well as autoimmune diseases recently reviewed [69,70]. Evidence from IBD patients demonstrated the essential roles of effector CD4+ T-cells, namely the Th1 and Th17 lymphocytes, upon inflammation [71]. Based on the presented in vivo and in vitro studies, D3 signaling in CD4+ T balanced effector lineages and favored the inflammatory potential of CD4+ T-cells during chronic experimental colitis. A D3R deficiency in CD4+ T-cells reduced their differentiation towards the Th1 phenotype and exacerbated the generation of Th2 cells, yet left unaltered Th17 differentiation [40]. Additionally, in IBD, Treg lymphocytes were also observed to be dysfunctional [72]. Interestingly, D3R signaling in Treg cells attenuated their suppressive activity and reduced their gut tropism. A D3R deficiency reduced inflammatory colitis manifestation in mice, and the inhibition of D3R signaling in the cells induced a potent anti-inflammatory effect [39]. Furthermore, the D5R receptor was observed to be highly expressed in colonic macrophages. D5R signaling suppressed the development of experimental colitis by regulating the balance of colonic M1/M2 macrophages, specifically the inhibition of M1 and the promotion of the IL-4/IL-13-triggered M2 macrophage polarization, respectively. A D5R deficiency indeed worsened experimental colitis [38]. D5R signaling also promoted gut tropism in CD4+ T-cells, and a D5R deficiency in those cells attenuated the development of inflammatory colitis and reduced cell infiltration into the gut mucosa without affecting T-cell differentiation, survival, or proliferation. The D5R deficiency intensified the expression of the gut-homing receptor CCR9 as well. However, D5R was discovered to be assembled with CCR9 to generate the gut-homing receptor, a cell surface complex that led the migration of CD4+ T-cells into the inflamed colonic mucosa. Thus, the disruption of the CCR9:D5R heteromer might be an attractive therapeutic strategy to mitigate CD4+ T-cell recruitment into the intestinal mucosa during inflammation. Most importantly, the number of CCR9:D5R clusters per lymphoid cell was higher in the inflamed mucosa obtained from IBD patients when compared with non-inflamed mucosae, with a significantly higher density of the clusters in CD patients [27]. It should be also mentioned in this context that the D2R *TaqIA* polymorphism (*rs1800497*) which confers a decreased receptor density was evaluated, and although the frequencies of the A1A1 and *A2A2* genotypes were similar among CD patients, UC patients, and healthy controls, the CD carriers of the *A2A2* genotype showed a lower risk for the development of refractory CD than A1A1 and A1A2 carriers [30]. Interestingly, a recent meta-analysis by You et al. (2019) suggested a significant association between the *D2R TaqIA* polymorphism and PD in the recessive and additive genetic models, especially in Caucasians [73]. Pharmacologically, DA has a 10-time higher affinity for D5R than for D1R, and a 20-time higher affinity for D3R than for D2R [68], and thus can exert either immunosuppressive or pro-inflammatory roles depending on either its high or low tissue concentrations, respectively. However, different factors including diet, microflora, medications, genotypes, or health conditions can affect its tissue concentration. The dynamics of such fluctuations remain unclear. Examples of DA levels in healthy and diseased intestines are given in Table 5.

Gut microbiota indeed play a considerable role in the generation of free catecholamines in the gut lumen [18,38,44]. Yet, no particular bacteria strains have been specifically associated with DA production. In the latest multi-omics study, the unexpected stability of the relative abundance of *Prevotella copri* in individuals with IBD was found [80]. Interestingly, D5R-deficient single-housed and co-housed mice were found to have a higher abundance of colitis-associated bacteria, such as Prevotellaceae and Clostridia_UCG-014, and a lower abundance of protective bacteria, such as Bacteroidaceae and Tannerellaceae [37].

The existing data from animal models [27,38,41,42,43] together with case reports [32,33,34,35,36,37] support the need to clarify if adjuvant therapy with L-DOPA on selective dopamine agonists would positively impact on the progression of IBD. CD and UC are both chronic relapsing and remitting IBDs of complex etiopathogenesis [80,81]. IBD variably impacts on individual patients’ quality of life and accounts for significant costs to societies, such as the costs of medications, hospitalizations, and absences from work, due to its typical onset between 18 and 35 years of age [82,83]. A recent study (2018) ranked IBD as the 5th most costly gastrointestinal condition in the USA for annual healthcare expenditures [84]. According to a British single center retrospective study from 2004, hospitalization affected a minority (around 14%) of patients but was responsible for half of the total direct healthcare system costs [85]. Recently, disease management with expensive biologic therapies has created better outcomes for patients [83]. Yet, in a short-term Dutch study, anti-TNF medications accounted for two thirds of the direct costs in CD (with a 3-month total cost of 1626 Euros) and one third in UC (with a 3-month total cost of 595 Euros) [86]. Still, up to 40% of patients do not respond well to the initial treatment [87]. Since the prevalence of these diseases continues to rise globally, especially in newly industrialized countries [88], healthcare systems should be prepared for an increasing IBD-related financial burden [82,83]. There is also a need to thoroughly verify if patients receiving dopamine antagonists, such as haloperidol, could be at greater risk of IBD development. For example, a review of the French pharmacovigilance database reported the intestinal necrosis associated with antipsychotics to be very rare, yet with high mortality and rapid worsening towards septic shock in spite of mild clinical symptoms such as abdominal pain associated with vomiting and/or diarrhea [89]. On the other hand, the elevated incidence and prevalence of psychiatric disorders in the IBD population [90] might be a confounding factor in such an analysis.

The development and progression of IBD involves several signaling pathways interacting simultaneously, with an unmet need for unequivocally efficient treatment. TH is a rate-limiting enzyme for norepinephrine and epinephrine as well, and α- and β-adrenergic receptors are present on nearly all types of immune cells [91]. NE at low concentrations (10^−9^ to 10^−7^ M) binds to α-adrenergic receptors with pro-inflammatory properties, while in higher concentrations (10^−7^ to 10^−5^ M) it expresses more affinity to β-adrenergic receptors with anti-inflammatory properties [48,92]. An increased risk of IBD in Caucasian populations was observed in association with the C-to-T single nucleotide polymorphism in *DβH* at position 1619 (*rs6271*), resulting in a *Arg549Cys* substitution [28]. *DβH* expression was also up-regulated in LPMCs from CD and UC patients [29]. Moreover, according to animal studies, a2-adrenoreceptor related pathways could play an important role in psychological stress-induced colitis, and thus a2-adrenoceptor antagonists might represent a potential therapeutic approach for the management of colitis [29,45]. The intact sympathetic innervation of the colon is required to properly regulate the intestinal myeloid compartment [48]. Yet, it should be noted that the experimental evidence also suggests a cross-activation of each other’s receptors by DA and NA (for a review, see [93]), which adds another layer of complexity to gastrointestinal immune regulation.

Despite the increasing data on the role of gastrointestinal DA in IBD, several knowledge gaps still exist, especially with regard to the direct influence of DA on the gut-to-brain signaling. The evidence regarding its role in the regulation of the intestinal mucosal barrier, its interplay with luminal microbiota, and the detailed localization of DA receptors in gastrointestinal wall is undoubtedly in its infancy. Conversely, psycho-neuro-endocrine-immune modulation through the brain-to-gut axis has a well-recognized role in the pathogenesis of IBD [94], and central dopaminergic circuits are much better described in comparison with the peripheral circuits, especially gastrointestinal ones. Our understanding of gastrointestinal dopaminergic neurotransmission in IBD is mostly based on animal models, yet those studies, including the revised studies, are prone to bias. To make matters worse, current scientific trends hugely discourage replication studies. In general, the number of animals/samples (frequently no more than 4–6 per experimental group) was limited and disproportional across experimental groups. Macroscopic scoring, histological analyses, and myeloperoxidase activity in the intestinal tissue were often used for the primary evaluation of the applied, yet different, animal models. Wild-type as well as knock-out mice of different strains were used in the majority of studies, and the applied models induced different phenotypes of experimental colitis. Due to different experimental setups and applied methodologies (Table 2), including, for example, a wide variety of antibodies used for various immunostaining techniques (Table 4), or even missing data from control groups or wild-type animals, a meta-analysis could not be performed. Numerical data was extracted from human studies only where the trends were cited (except from intestinal DA levels which were compiled in Table 5). From the pathophysiological point of view, it should also be stressed that none of the existing IBD animal models entirely replicate the human disease, and only some of them (the adoptive T-cell transfer model among those reviewed above, see Table 2) induced inflammation in the small intestine [95]. Consequently, results from animal and human studies were clearly separated.

Still, the pathogenesis of IBD and PD is far from understood. Apart from already recognized epidemiological [7] and genetic [6] relationships between the diseases, the T-cell driven inflammation, possibly due to catecholaminergic impairments, represents another link [96]. According to the reviewed evidence, *Rag1−/−* mice, lacking T- and B-lymphocytes and treated with 6-OHDA exhibited histological features of colitis, which were further exacerbated by surgical (instead of chemical) sympathectomies [48], while WT mice did not develop colitis after a sympathectomy [97]. Such chemical sympathectomies also improved TNBS-induced colitis [50], and so did the MPTP destruction of peripheral dopaminergic neurons in IA-treated rats [49]. Thus, intact dopaminergic and adrenergic neurotransmission is required for mucosal immune homeostasis. Gastrointestinal immune activation modulates the gut-to-brain axis, as evidenced, for example, by an exacerbation of α-synuclein pathology in the brains of transgenic mice with DSS-induced colitis [98]. The important role of systemic inflammation in the pathogenesis of both IBD and PD is emphasized by the fact that an early exposure to anti-inflammatory anti-TNF therapy among IBD patients was associated with substantially reduced subsequent PD incidence [99]. On the other hand, intestinal inflammation was observed in a rodent model of 6-OHDA-induced nigrostriatal neurodegeneration, suggesting a bidirectional link between gastrointestinal inflammation and brain neurodegeneration [100,101]. 

## 4. Materials and Methods

A systematic search was conducted according to the Preferred Reporting Items for Systematic reviews and Meta-Analyses (PRISMA) methodology [102]. The MEDLINE, Cochrane, Web of Science, and Scopus databases were searched from their earliest records to 31 July 2021. The following search terms were applied for the study: dopamine, dopaminergic, dopamine agonist, dopamine antagonist, inflammatory bowel disease, ulcerative colitis, and Crohn’s disease. All duplicates were removed using the Mendeley Reference Manager (licensed for Jagiellonian University, Poland) and furthermore, all articles were manually and independently screened by all authors. The criteria for eligibility consisted of: English language, original article type (including case reports), full text availability, and adherence to the research topic. Additionally, a reference search was performed. The final list of full texts was double-checked and the data was extracted independently by all investigators blinded to each other. It should be noted that we primarily focused on the role of peripheral dopaminergic neurotransmission in IBD. However, animal models associated with sympathetic denervation together with disease specific models (usually written in the context of Parkinson’s disease) were also included into the analysis but only the peripheral perspective was taken into account. Both human and animal studies that focused on the research topic were taken into account; however, their results were grouped separately. In vitro data was extracted from all the studies, yet we did not look for additional studies applying in vitro models alone. The following databases: Clinical Trials (http://clinicaltrials.gov, accessed on 31 July 2021), EU Clinical Trials Register (https://www.clinicaltrialsregister.eu, accessed on 31 July 2021), Australian New Zealand Clinical Trials Registry (https://www.anzctr.org.au, accessed on 31 July 2021), and OpenTrials (https://explorer.opentrials.net/, accessed on 31 July 2021), were also searched for the ongoing clinical trials with dopaminergic compounds in IBD patients.

## 5. Conclusions

**Figure 3:**
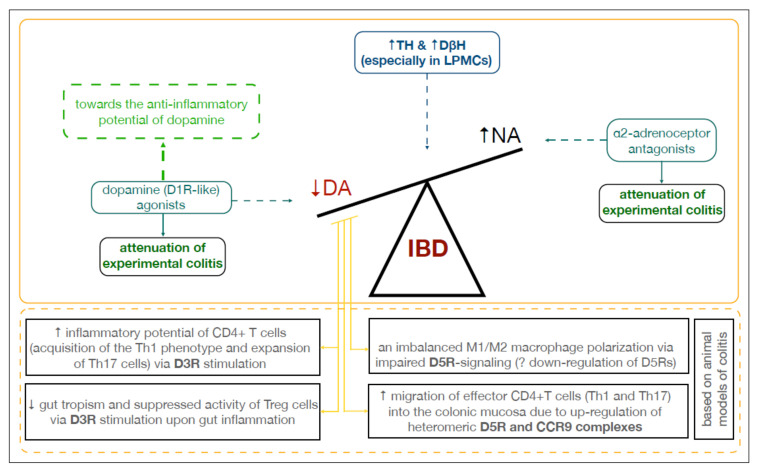
Dopamine in the inflamed colon. **Abbreviations:** CCR9: C-C chemokine receptor type 9; DA: dopamine; D3R, D5R: dopamine receptors; DβH: dopamine β-hydroxylase; IBD: inflammatory bowel disease; LPMCs: lamina propria mononuclear cells; NA: noradrenaline; TH: tyrosine hydroxylase.

Collectively, this systematic review underlined the undisputed and complex role of DA in the development and progression of IBD, and especially its direct role in gastrointestinal immune maintenance upon inflammation (Figure 3). However, limited attention has been paid to the neuro-immune interactions, which could further link IBD and PD pathogenesis throughout the intestinal wall. Methodological limitations of the above-mentioned preclinical studies were also exposed. Our understanding of the distribution of dopamine receptors and the role of the dopaminergic system in the gastrointestinal tract is far behind the advancements in the central nervous system. Thus, further research is inevitably needed in order to know how to utilize gastrointestinal DA-rich cells to cope with gut and brain inflammation. Above all, more observational studies should be encouraged to support the already existing preclinical data relating to IBD.

## Figures and Tables

**Table 1:** Clinical characteristics of the observational studies and case reports included into the analysis. Abbreviations: CD: Crohn’s disease; N/A: not available; UC: ulcerative colitis.

Cross-Sectional Studies					
References	IBD Patient Characteristics (Including the Number of Patients/Samples)	Inclusion Criteria	Exclusion Criteria	Control Subject Characteristics (Including the Number of Patients/Samples)	Type of Samples
Osorio-Barios et al., 2021 [27]	n = 3 with UC, n = 4 with CD (inflamed mucosa)	N/A	N/A	n = 6 (noninflamed mucosa)	Intestinal biopsies from HUB-ICO-IDIBELL Biobank
Gonzalez-Lopez et al., 2019 [28]	In total, n = 45 (38.2 ± 3.8 years): n = 21 (10 females) with CD and n = 24 (12 females) with UC (from Penn State Hershey Medical Center’s IBD BioBank for Central Pennsylvania	Endoscopic criteria	N/A	n = 74 (65.1 ± 8.7 years old), free of any diagnosed major neurological illness or IBD	Blood samples
Bai et al., 2015 [29]	n = 3 with active CD, n= 4 with active UC	IBD diagnosis based on clinical, endoscopic, histological, and/or radiological criteria (Bernstein et al., 2010)	N/A	n = 4	Colonic biopsies
Magro et al., 2006 [30]	n = 143 with CD (81% were on mesalamine, 21% on corticosteroids, 45% on azathioprine and/or infliximab), n = 77 with UC (72% were on mesalamine, 22% on corticosteroids, 19% on azathioprine)	Refractory treatment for CD: (1) moderate to severe CD in patients taking mesalamine for at least 2 months and corticosteroids for at least 1 month; (2) patients with moderate to severe CD taking mesalamine for at least 2 months and were unable to reduce corticosteroids for at least 2 months in two attempts (corticosteroid dependency); (3) patients with moderate to severe CD taking azathioprine for at least 4 months; (4) all patients with moderate to severe CD taking corticosteroids for at least 1 month or azathioprine for at least 4 months and who needed infliximab for disease control; (5) patients with active perianal CD that has been treated with antibiotics for at least 2 months; (6) patients with relapsing perianal CD; (7) patients with active perianal CD in treatment with antibiotics and azathioprine for at least 4 months and who needed infliximab for CD control. Refractory treatment for UC: (1) moderate to severe UC in patients taking mesalamine for at least 2 months and corticosteroids for at least 1 month; (2) patients with moderate to severe UC taking mesalamine for at least 2 months who were unable to reduce corticosteroids for at least 2 months in two attempts (corticosteroid dependency); (3) patients with moderate to severe UC taking azathioprine for at least 4 months, (4) all patients with moderate to severe UC taking corticosteroids for at least 1 month and/or azathioprine for at least 4 months and who needed cyclosporine for disease control; (4) all patients with severe UC taking corticosteroids and who needed cyclosporine for disease control.	N/A	n = 93 (random blood donors)	Blood samples
Magro et al., 2002 [31]	n = 22 with CD, n = 21 with UC, all patients had a disease history of several years; all but one patient were on mesalazine, none were on steroids or azathioprine	Endoscopic criteria	N/A	n = 16 with normal colonoscopy (subjects with abdominal pain or colonic polyps)	Biopsy specimens, 10 for each patient, taken from inflamed and noninflamed colonic mucosae. The specimens obtained from IBD patients in noninflamed tissues were taken 10 cm from the inflamed area, the biopsies in the inflamed colonic mucosa were not taken from ulcers, and control biopsies were taken at 20 cm from the anal margin
**Case Reports**					
**References**	**Patient Characteristics**				**Successful treatment**
Tomic et al., 2015 [32]	63-year-old man with CD and PD, admitted to an outpatient clinic for continuous duodenal levodopa infusion. Considerable clinical improvements without any complications were noted during an 8-month follow up.				Continuous duodenal levodopa (L-DOPA) infusion
Check et al., 2011 [33]	39-year-old woman with a 12-year history of CD that was nonresponsive to adalimumab, mesalamine, prednisone, cyclophosphamide, and infliximab.				Dextroamphetamine (induces the release of dopamine within the mesocorticolimbic system)
Kane et al., 2003 [34]	Two women with Crohn’s ileitis treated with bupropion daily for smoking cessation. Another 2 patients with Crohn’s colitis were treated with bupropion for depression and similarly experienced a remission of their disease symptoms.				Bupropion (a weak inhibitor of the neuronal uptake of norepinephrine and dopamine)
Kast et al., 2001 [35]	44-year-old woman with 10-year history of CD, reporting daily pain, frequent blood in stool, and inadvertent loss of stool several times a month. Treated with bupropion due to dysthymia.				
Kast 1998 [36]	A 33-year-old woman with an 18-year history of CD presented for treatment of a major depressive episode. Her depression responded well, and all signs of abdominal discomfort were absent for the first time in her adult life after phenelzine treatment initiation				Phenelzine (monoamine oxidase inhibitor)
Lechin et al., 1982 [37]	35-year-old woman, 48-year-old man, and 13-year-old boy, all with diagnosed ulcerative colitis, reported developing cramping abdominal pain and bloody diarrhea. The corticosteroid treatment was unsuccessful (2 patients) or partly successful (48-year-old man).				Thioperazine (an antagonist on dopaminergic (D1, D2, D3, and D4), serotonergic (5-HT1 and 5-HT2), histaminergic (H1), α1/α2, and cholinergic (M1/M2) receptors)

**Table 2:** Animal studies included in the analysis. Abbreviations: 6-OHDA: 2,4,5-trihydroxyphenethylamine; DAI: disease activity index; DNBF: 2,4-dinitrofluorobenzene; DSS: dextran sulphate sodium; IA: iodoacetamide; MPO: mieloperoxidase; MPTP: 1-methyl-4-phenyl-1,2,3,6-tetrahydropyridine; TNBS: 2,4,6-trinitrobenzene sulfonic acid; routes of administration: i.p.: intraperitoneal; i.g.: intragastric; i.v.: intravenous; s.c.: subcutaneous.

Disease-Specific Models					
References	Experimental Model	Pharmacological/Biological Intervention	Animals	Time Frame	Assessment of Colonic Inflammation
Liu et al., 2021 [38]	2.5% DSS (TdB Consultancy, Sweden) as an addition to drinking water for 6 days	SKF-38393 (Tocris, UK) given via i.p. (10 mg/kg daily) starting 1 d before DSS	Wild-type C57BL/6 male mice, Drd5−/− mice (Cyagen Biosciences Inc, China); Rosa26-tdTomato and Cx3cr1-Cre mice (provided by Dr. Jiawei Zhou, Institute of Neuroscience, Chinese Academy of Sciences, China)	Up to 6 weeks	DAI and histological scoring
Osorio-Barrios et al., 2021 [27]	1.75% DSS (MP Biomedical, USA) as an addition to drinking water for 5 days; Rag 1−/− mice received via i.p. 5x 105 naive CD4+ T-cells	CD4+ T-cells (6 × 106 total cells per mouse) bearing single positive congenic markers (CD45.1+, CD45.2- or CD45.1-, CD45.2+)	Wild-type C57BL/6 (Drd5^+/+^, Cd45.2^+/+^), Ccr9−/−, Rag1−/−, (Jackson Laboratory), C57BL/6 Drd5−/− (donated by Dr. Sibley), B6.SJL-Ptprca (CD45.1+) (donated by Dr. Rosa Bono), Drd5−/−Cd45.1+/+ as well as Cd45.1+/–Cd45.2+/– (generated by crossing parental strains) mice, 6–10 weeks old	Up to 12 weeks	Body weight, DAI, immune cell infiltration
Ugalde et al., 2021 [39]	1 or 1.75% DSS (MP Biomedicals, USA) as an addition to drinking water for 8 days, or 2.5 mg OVA (Genescript, USA) given via oral lavage for 5 days	Drd3-deficient Treg cells (CD3+, CD4+, CD25+ cells, 3 × 105 cells per mouse), ex vivo RV-shDrd3-transduced (retroviral vectors codifying for an shRNA directed to reduce Drd3 transcription, 2 × 105 cells per mouse), both given via i.v.	Wild-type C57BL/6 (Drd3+/+, Cd45.2+/+), Ccr9−/−, Rag1−/−, C57BL/6 Foxp3gfp (Jackson Laboratory), Rag1−/−Drd3−/−, Cd45.1+/−Cd45.2+/−, Foxp3gfp Drd3−/−, and Ccr9−/−Drd3−/− (generated by crossing parental strains) mice, 8–10 weeks old	Up to 10 weeks	DAI and histological scoring
Contreras et al., 2016 [40]	Rag1−/− mice received via i.p. 5 x 105 naive (CD45RBhigh) or regulatory/memory (CD45RBlow) CD4+ T-cells via i.p; 100 mg OVA in CFA via s.c. or 20 mg OVA in Alum adjuvant via i.p. (Sigma Aldrich, St. Louis, MI, USA; Thermo Scientific, Waltham, MA, USA)	Naive CD45.2+, Drd3+/+, or Drd3−/− OT-II (105) cells i.v. transferred before s.c. immunization with OVA; a mixture (107) of total Drd3+/+ or Drd3−/− OT-II CD4+ T-cells and WT CD4+ T-cell-depleted splenocytes (in a 15:85 ratio) given via i.v. before i.p. immunization with OVA	Wild-type C57BL/6, Rag1−/− (Jackson Laboratory), Drd3−/−, (donated by Dr. Caron), OT-II, B6.SJL-Ptprca (CD45.1+) (donated by Dr. Rosa Bono), Drd3−/− OT-II (generated by crossing parental strains) mice, 6–10 weeks old	Up to 10 weeks	
Oehlers et al., 2016 [41]	0.5% DSS or TNBS (final concentration 70 ug/mL) as an addition to the larval media	Devazepide (Tocris, UK), dexamethasone, haloperidol, cabergoline, lorglumide, and sincalide (Sigma-Aldrich, USA) as an addition to the larval media	Zebrafish embryos obtained from natural spawnings and raised at 28.5 °C in E3 embryo medium supplemented with methylene blue until 1 dpf	Up to 6 dpf	Neutrophil enumeration
Bai et al., 2015 [29]	TNBS (1 mg, Sigma Aldrich, USA) given rectally; restrain stress model	RX821002 (250 ug per mouse) given via i.p.	Male BALB/c mice 7–8 weeks old, weighing approximately 22 g	10 days	Body weight and histological scoring
Kawano et al., 2015 [42]	4% DDS (MP Biomedicals, USA) in drinking water for 4 days	Berberine (high purity extract from Coptis rhizome provided by Tsumura & Co, Japan), SCH23390, L750667, and lipopolysaccharide (Sigma-Aldrich, USA) given via i.p.	C57BL/6 mice (Japan SLC, Japan)	7 days	Body weight, colon length, and histological scoring
Tolstanova et al., 2015 [43]	6% IA (Sigma, USA) given rectally and IL-10-deficient colitis	Quinpirole (Sigma, USA) and cabergoline (Pfizer, USA), both given via i.g.	Female Sprague–Dawley (Harlan Laboratory, San Diego, USA) rats (170–200 g), female Wistar (Animal Research Facility, KNU, Ukraine) rats (170–220 g), IL-10 knockout mice on a C57BL/6J background (12 weeks old), sex- and age-matched wild-type C57BL/6J (Jackson Laboratory, USA) mice	Up to 12 weeks	DAI, histological scoring, MPO activity
Rooks et al., 2014 [44]	TRUC colitis (T-bet−/−Rag2−/−)	Metronidazole (1 g/L, Sigma Aldrich, USA), gentamicin (2 g/L, Cellgro, USA), vancomycin (500 mg/L, Sigma Aldrich, USA), dissolved in drinking water; a hamster anti-mouse TNF-a neutralizing antibody (15 mg/kg, clone TN4-19.12, Bio X Cell, USA) given via i.v., FACS-sorted peripheral lymph node CD4+CD62LhiCD25+ cells (75 000 cells per mouse) given via i.v.	Homozygous T-bet−/−Rag2−/−(TRUC) SPF BALB/c mice and genobiotic TRUC BALB/c mice (Harvard School of Public Health, USA)	Up to 8 weeks	Histological scoring
Bai et al., 2009 [45]	5% DSS (Amersham Pharmacia Biotech AB, Sweden) in drinking water every second day or TNBS (2.5 mg, Sigma Aldrich, USA) given rectally	RX821002 (10 mg/kg) or UK14304 (2 mg/kg), both given via i.p.	Male BALB/c mice 7–8 weeks old, weighing approximately 22 g	Up to 10 days	DAI, histological scoring, MPO activity
Magro et al., 2004 [46]	TNBS (30 mg; Sigma Aldrich, USA) given rectally	-	Male Wistar rats (Harlan-Interfauna, Spain) weighing 220–250 g	7 days	Macroscopic and histological scoring, MPO activity
Herak-Perkovic et al., 2001 [47]	0.5% DNBF (Sigma Aldrich, USA) given rectally	Domperidone (Sigma Aldrich, USA) or bromocriptine (Bromergon, Slovenia), both given via i.p.	Male BALB/c mice (Institute Ruder Boskovic, Croatia), 8–12-week-old (20–25 g)	5 days	Histological scoring
**Models Associated with Sympathetic Denervation Together with Disease-Specific Models**					
Willemze et al., 2019 [48]	6-OHDA (80 mg/kg body weight on three consecutive days, and every 10 days thereafter; Sigma) given via i.p.; intestine-specific sympathectomy (transection of the superior mesenteric nerve along the mesenteric artery)	-	Female C57BL/6 inbred mice (8–12 weeks old; Charles River Laboratories, Netherlands), male and female Rag1−/− mice (8–12 weeks old; The Jackson Laboratory, USA), male and female Rag1−/−Adrβ2−/− mice (Philippe Blancou at the Institute of Molecular and Cellular Pharmacology, France)	Up to 4 weeks	Macroscopic, histological, and endoscopic scoring
Prysiazhniuk et al., 2017 [49]	MPTP (4 × 20 mg/kg, Sigma Aldrich, USA) given via s.c. and/or 6% IA (rectally)	-	Male Wistar rats, 170–200 g	Up to 18 days	DAI, macroscopic and histological scoring, MPO activity, phagocytosis assay
McCafferty et al., 1997 [50]	TNBS (30 mg, ICN Pharmaceuticals, USA; Fluka, Canada) given rectally and/or 6-OHDA (total dose 300 mg/kg, RBI, USA) given via s.c.	Capsaicine (AstraPharma, intracolonic, or via s.c.), lidocaine (USP, intracolonic)	Male or female Sprague-Dawley rats (Charles River Breeding Farms, Canada) weighing 250–300 g	NA	Body weight, mortality, macroscopic and histological scoring, MPO activity

**Table 3:** Brief characteristics of compounds with affinity to dopamine and adrenergic receptors applied in the reviewed in vivo studies, based on the open chemistry PubChem database (https://pubchem.ncbi.nlm.nih.gov) as well as other directly linked databases (accessed on 10 and 18 August 2021). Abbreviations: DR: dopamine receptors; N/A: not available.

	Bromocriptine	Cabergoline	Quinpirol	SKF-38393	Berberine	Domperidone	Haloperidol	Brimonidine (UK14304)	RX821002
IUPAC name	6aR,9R)-5-bromo-N-[(1S,2S,4R,7S)-2-hydroxy-7-(2-methylpropyl)-5,8-dioxo-4-propan-2-yl-3-oxa-6,9-diazatricyclo [7.3.0.02,6]dodecan-4-yl]-7-methyl-6,6a,8,9-tetrahydro-4H-indolo[4,3-fg]quinoline-9-carboxamide	(6aR,9R,10aR)-N-[3-(dimethylamino)propyl]-N-(ethylcarbamoyl)-7-prop-2-enyl-6,6a,8,9,10,10a-hexahydro-4H-indolo[4,3-fg]quinoline-9-carboxamide	(4aR,8aR)-5-propyl-1,4,4a,6,7,8,8a,9-octahydropyrazolo[3,4-g]quinoline	5-phenyl-2,3,4,5-tetrahydro-1H-3-benzazepine-7,8-diol	16,17-dimethoxy-5,7-dioxa-13-azoniapentacyclo[11.8.,0.02,10.04,8.015,20]henicosa-1(13),2,4(8),9,14,16,18,20-octaene	6-chloro-3-[1-[3-(2-oxo-3H-benzimidazol-1-yl)propyl]piperidin-4-yl]-1H-benzimidazol-2-one	4-[4-(4-chlorophenyl)-4-hydroxypiperidin-1-yl]-1-(4-fluorophenyl)butan-1-one	5-bromo-N-(4,5-dihydro-1H-imidazol-2-yl)quinoxalin-6-amine	2-(3-methoxy-2H-1,4-benzodioxin-3-yl)-4,5-dihydro-1H-imidazole
Molecular formula	C_32_H_40_BrN_5_O_5_	C_26_H_37_N_5_O_2_	C_13_H_21_N_3_	C_16_H_17_NO_2_	C_20_H_18_NO4^+^	C_22_H_24_ClN_5_O_2_	C_21_H_23_ClFNO_2_	C_11_H_10_BrN_5_	C_12_H_14_N_2_O_3_
Molecular weight [g]	654.6	451.6	219.33	255.31	336.4	425.9	375.9	292.13	234.25
Affinity to catecholaminergic receptors	Primarily a D2R subfamily agonist (higher affinity to D2/3R)	A long-acting D2R agonist	A D3/D4R agonist	D1R subfamily agonist (a higher affinity to D5R)	A D1/D2R antagonist	A D2/D3R peripheral-specific antagonist	A D2R subfamily antagonist	A highly selective a2-adrenoceptor agonist	An a2-adrenoceptor antagonist
Biological half life	2–8 h	63–69 h	N/A		N/A	7 h	14.5–36.7 h	3 h (ocular administration), 12 h (topical administration)	N/A
Protein binding	90–96%	40–42%				91–93%	88.4–92.5%	N/A	
Metabolism in human subjects	P450 3A4 and excreted primarily in the feces via biliary secretion.	Extensively metabolized, predominately via hydrolysis of the acylurea bond of the urea moiety, and cytochrome P-450 mediated metabolism appears to be minimal			Extensive metabolism after oral administration	CYP1A2, CYP2B6, CYP2C8, CYP2D6, CYP3A4	Highly lipophilic and is well-absorbed from the gastrointestinal tract. However, the first-pass hepatic metabolism decreases its oral bioavailability; reduced CYP2D6 enzyme (including genetic polymorphisms) activity may result in increased concentrations	It was reported to be metabolized in the cornea after topical administration reaches systemic circulation and undergoes extensive hepatic metabolism mediated by hepatic aldehyde oxidases	
Drug indication	Galactorrhea due to hyperprolactinemia, prolactin-dependent menstrual disorders and infertility, prolactin-secreting adenomas, prolactin-dependent male hypogonadism, Parksinsonian syndrome, and as an off-label medication to treat restless legs syndrome and neuroleptic malignant syndrome	High prolactin levels, prolactinomas, Parkinson’s disease (but also possesses antioxidant and neuroprotective properties due to its free radical scavenging activity)	Research chemical		Parasitic and fungal infections, diarrhea (dietary supplements); a research chemical with potential antineoplastic, radiosensitizing, anti-inflammatory, anti-lipidemic, and antidiabetic activities	Dyspepsia, heartburn, epigastric pain, nausea, vomiting	Schizophrenia, tics and vocal utterances of Tourette’s disorder, severe behavioral problems in children of combative, explosive hyperexcitability and short-term treatment of hyperactive children with excessive motor activity with accompanying conduct disorders	Lowering intraocular pressure in patients with open-angle glaucoma or ocular hypertension as a monotherapy or in combination; persistent (non-transient) facial erythema of rosacea in adults 18 years of age or older	Research chemical
Toxicity/side effects	Nausea, headache, vertigo, constipation, light-headedness, abdominal cramps, nasal congestion, diarrhea, severe hypotension	Nasal congestion, syncope, hallucinations	N/A			Galactorrhea, gynecomastia, menstrual irregularities	Extrapyramidal reactions, hypotension, respiratory difficulty, impairment of consciousness	Hypotension, asthenia, vomiting, lethargy, sedation, bradycardia, arrhythmias, miosis, apnea, hypotonia, hypothermia, respiratory depression, and seizures	N/A

**Table 4:** Dyes and antibodies used for in vivo and in vitro histological analysis and/or immunostaining (immunohistochemistry, immunoblotting, flow cytometry, enzyme-linked immunosorbent assay/ELISA).

Disease-Specific Models		
References	Chemical Dyes for Histology	Primary Antibodies for Immunostaining (Including Immunohistochemistry, Flow Cytometry, Western Blotting, and ELISA)
Liu et al., 2021 [38]	Hematoxylin and eosin	Anti-Inos (ab15323, Abcam, UK), -β-actin (A1978, Sigma), -Arg1 (PA585267), -F4/80 (14-4801-82), -PE IgG (35- 4914-81), -PEcy7 IgG 25-4914-82 (eBioscience, USA); anti-phosphorylated PKA C (5661, SCT); anti-PKA C-α (55388-1-AP), -Drd5 (20310-1-AP/ADR-005, Proteintech/Alomone); anti-phosphorylated IKKα/β (2697), -IKKβ (2370), -phosphorylated IκBα (9246), -phosphorylated CREB (9198s, Cell Signaling Technology); anti-CREB (48601-2, SAB), anti-TCR-β-eFlour450 (48-5961-80), -CD45-AF700 (30-F11, 85-11-0112-81), -APC-CD45.1 (17-0453-82), -APC-eflour-780-CD45.2(47-0454-82), -CD11b- FITC (M1/70,85-12-0114-81), -F4/80-APC (BM8, 17- 4801-82), -Ly6c-PE-Cy7 (HK1.4, 25-5932-82), -Ly6g-percpcy5.5 (48-9668-82), FVD-eFlour^®^506 (65- 0866), -APC-TNFα (17-7321-82, eBioscience, USA); anti-CD11c (12-0114-82), -CD19 (17- 0193-80), -NK1.1-PE-cy7 (25-5941-81, Thermo)
Osorio-Barrios et al., 2021 [27]	Zombie Aqua (Biolegend, USA)	Anti-CCR9 (1:100 dilution, ab140765, Abcam, UK), anti-DRD5 (1:100 dilution, 324408, Calbiochem, USA); anti-IFNγ (clone XMG1.2), -a4b7 (clone DATK32), -CCR9 (clone CW.1.2, eBioscience, USA); anti-CD4 (clone GK1.5), -CD25 (clone PC61), -CD44 (clone IM7), -CD62L (clone MEL14), -IL-17A (clone TC11-181710.1), -CD45.2 (clone 104), -CD45.1 (clone A20), -TCR b chain (clone H57-597) (BioLegend, USA)
Ugalde et al., 2021 [39]	Hematoxylin and eosin, Alcian blue, Zombie Aqua (Biolegend, San Diego, CA, USA), Cell trace violet (Invitrogen, USA)	Anti-FoxP3 (clone FJK-16S), -IFNγ (clone XMG1.2), -α4β7 (clone DATK32), -CCR9 (clone CW.1.2) (eBioscience, USA); anti-CD4 (clone GK1.5),-CD25 (clone PC61), -CD44 (clone IM7), -CD62L (clone MEL14), -IL-17A (clone TC11-181710.1), -CD45.2 (clone 104), -CD45.1 (clone A20), -TCRVα2 (clone B20.1), -TCRVβ5 (clone MR9-4), -CD28 (clone 37.51),-CD3ε (clone 145-2C11), -IFNγ (clone AN-18) (Biolegend, USA); anti-DRD3 (ADR-003) (Alomone labs)
Contreras et al., 2016 [40]	Hematoxylin and eosin	Anti-CD4 (GK1.5), -CD44 (IM7), -CD62L (MEL-14), -CD25 (PC61), -IL-7Ra (A7R34), -CD27 (LG.3A10), -CD45.2 (104), -CD45.1 (A20), -IFNγ (XMG1.2),-IL-17A (TC11-18H10.1),-T-bet (4B10),-IL-4 (11B11),-IL-5 (TRFK5),-CD3ε (145-2C11),-CD28 (37.51), –IFNγ (AN-18),–IL-4 (11B11),–IL-12 (C17.8), -mouse IgG1 (RMG1-1) (BioLegend, USA); anti-Foxp3 (FJK16s),-IL-13 (eBio13A),-IFNγ (XMG1.2) (eBioscience, USA); anti–IL-4 (BVD4-1D11, BVD6-24G2) (BD Biosciences, USA), anti-suppressor of cytokine signaling (SOCS) 3 (H-103), -SOCS5 (M-300, Santa Cruz Biotechnology, USA)
Oehlers et al., 2016 [41]	DAF-FM-DA, 4-amino-5-methylamino-2′, 7′- difluorofluorescein diacetate (Invitrogen, USA)	-
Bai et al., 2015 [29]	Hematoxylin and eosin	Anti-DBH antibodies (Santa Cruz Biotechnology, USA); anti-TNFα, -IL-1β (ELISA, R&D Systems, USA)
Kawano et al., 2015 [42]	Hematoxylin and eosin (New Histo. Science Laboratory Co, Japan)	Anti-IFNγ, -IL-6, -TNFα, -IL-1β, -IL-12, -IL-4, -TGFβ, -IL-23, -IL-17 (ELISA DuoSet kits, R&D Systems)
Tolstanova et al., 2015 [43]	Evans blue, fluorescein isothiocyanate-conjugated (FITC)–dextran, hematoxylin and eosin (Sigma, USA)	Anti-TH, -Akt, -phospho-AktSer473, -Src, -phospho-Src familyTyr416 (Cell Signaling Technology, USA), -dopamine transporter DAT, -D2-R, -β-actin (Santa Cruz, USA), -GAPDH (EnCor Biotech, USA), -Von Willebrand factor (Chemicon, USA)
Rooks et al., 2014 [44]	Hematoxylin and eosin	Anti-dopamine DOP Research ELISA (Labor Diagnostika Nord, Germany)
Bai et al., 2009 [45]		Anti-TH or anti-DBH antibodies (Santa Cruz Biotechnology, USA); anti-TNFα, -IL-1β (ELISA, R&D Systems, USA)
Magro et al., 2004 [46]	-	Anti-IFNγ (an assay kit according to van der Meide et al., 1990)
Herak-Perkovic et al., 2001 [47]	Hematoxylin and eosin	-
	**Models Primarily Associated with Sympathetic Denervation Together with Disease-Specific Models**	
Willemze et al., 2019 [48]	Hamatoxylin and eosin	Anti-IL-1β, -IL-4, -IL-6, -IL-10, -IL-17, -IFNγ and TNFα (R&D systems, UK); anti-human-IL-1β, -IL-6, -IL-10, -IL-12 and TNFα (CBA; BD Bioscience, USA); anti-CD45 (Brunschwig, USA); anti-CD11b, -Ly6G, -CD64, -MHC-II (Biolegend, USA); anti-CD11c, -Ly6C (Affymetrix, Austra)
Prysiazhniuk et al., 2017 [49]	-	Anti-TH and -β actin (Santa Cruz Biotechnology, Germany)
McCafferty et al., 1997 [50]	Hamatoxylin and eosin	-

**Table 5:** Dopamine levels (original values) in healthy and diseased intestinal tissue samples of rodents and human subjects measured by HPLC. Abbreviations: 6-OHDA: 2,4,5-trihydroxyphenethylamine; TNBS:2,4,6-trinitrobenzene sulfonic acid; CD: Crohn’s disease, UC: ulcerative colitis; * estimates obtained from graphs because no exact values were given in the text.

	Human	Rat	Mice
Duodenum	-	239 pmol/g [74]	-
	-	57 pmol/g **6-OHDA treatment** [74]	-
Jejunum	-	61 pmol/g (mucosa) [75]	0.03 pmol/g [76]
		41 pmol/g (mucosa) [77]	
		36 pmol/g (epithelial cells) [77]	
Ileum	-	30 pmol/g [46]	15.7 ng/g [18] (gut lumen)
	-	28 pmol/g **TNBS-treatment** [46]	-
		* 163 pg/µg [78]	
	-	* 167 pg/µg **6-OHDA treatment** [78]	-
Cecum	-	-	115.4 ng/g [18] (gut lumen)
Colon	* 135 pmol/g [30]	26 pmol/g [46]	177 ng/g [18] (gut lumen)
	* 50 pmol/g (inflamed mucosa) **in both CD and UC patients** [30]	17 pmol/g **TNBS-treatment** [46] in inflamed colonic mucosa	1.5 × 10^−7^ M [38]
	-	* 60 pg/µg [78]	1 × 10^−7^ M [38] **DSS treatment**
	-	* 130 pg/µg **6-OHDA treatment** [78]	-
	-	*12 pg/mg [79]	-
		* 27 pg/mg **6-OHDA treatment** [79]	-

## Data Availability

Not applicable.
